# ACE Model for Older Adults in ED

**DOI:** 10.3390/geriatrics4010024

**Published:** 2019-02-21

**Authors:** Martine Sanon, Ula Hwang, Gallane Abraham, Suzanne Goldhirsch, Lynne D. Richardson, GEDI WISE Investigators

**Affiliations:** 1 Icahn School of Medicine at Mount Sinai, Department of Geriatrics and Palliative Medicine 1, New York, NY 10029, USA; ula.hwang@mountsinai.org (U.H.); Suzy.goldhirsch@mssm.edu (S.G.); 2 Icahn School of Medicine at Mount Sinai Department of Emergency Medicine2, New York, NY 10029, USA; Gallane.abraham@mountsinai.org (G.A.); lynne.richardson@mountsinai.org (L.D.R.)

**Keywords:** geriatric emergency care, IDT, transitional care, ACE model

## Abstract

The emergency department (ED) is uniquely positioned to improve care for older adults and affect patient outcome trajectories. The Mount Sinai Hospital ED cares for 15,000+ patients >65 years old annually. From 2012 to 2015, emergency care in a dedicated Geriatric Emergency Department (GED) replicated an Acute Care for Elderly (ACE) model, with focused assessments on common geriatric syndromes and daily comprehensive interdisciplinary team (IDT) meetings for high-risk patients. The IDT, comprised of an emergency physician, geriatrician, transitional care nurse (TCN) or geriatric nurse practitioner (NP), ED nurse, social worker (SW), pharmacist (RX), and physical therapist (PT), developed comprehensive care plans for vulnerable older adults at high risk for morbidity, ED revisit, functional decline, or potentially avoidable hospital admission. Patients were identified using the Identification of Seniors at Risk (ISAR) screen, followed by geriatric assessments to assist in the evaluation of elders in the ED. On average, 38 patients per day were evaluated by the IDT with approximately 30% of these patients formally discussed during IDT rounds. Input from the IDT about functional and cognitive, psychosocial, home safety, and pharmacological assessments influenced decisions on hospital admission, care transitions, access to community based resources, and medication management. This paper describes the role of a Geriatric Emergency Medicine interdisciplinary team as an innovative ACE model of care for older adults who present to the ED.

## 1. Introduction

The emergency department (ED) setting can be an extremely challenging environment for vulnerable patients, especially those with advanced age, baseline cognitive impairment, functional limitations, or frailty. While the ED is the traditional entry point into the healthcare system providing essential acute emergency medical care, it is often not an ideal care environment for many older, vulnerable patients. Older adults tend to have greater complex medical co-morbidities, higher illness severity, and limited cognitive and functional reserves to overcome the stress of an acute illness and a hospitalization [1,2,3,4]. 

While the reasons for presenting to the ED are varied, there is a growing recognition that for older adults an ED visit is a critical opportunity to identify and intervene upon unique care needs in addition to the acute illness [2,3,4,5]. 

### 1.1. Geriatric Emergency Department

The traditional model of ED care prioritizes efficient triage and treatment for an acute illness or trauma [4]. This model of rapid ED care does not afford time often required to identify and address the complexities of frail and vulnerable older adult patients with multiple chronic conditions complicated by functional, cognitive, and psychosocial challenges [6]. Patients with these multiple co-morbidities, however, have higher disease and symptom burden which must be addressed to ensure successful care of the acute medical condition. Comprehensive evaluations and disposition plans that account for the functional, cognitive, and psychosocial issues that impact health care-related quality of life are essential to the successful management of the acute medical condition [6,7].

Recognizing the need for a better approach to older patients in the emergency room, Mount Sinai Hospital opened a dedicated Geriatric Emergency Department (GED) in 2012, transforming the way emergency care is provided for older adults. Taking advantage of a strong geriatrics presence in the hospital, and enthusiastic buy-in from the Emergency Medicine Department, this innovative GED model included universal and focused assessments on common geriatric syndromes and daily comprehensive interdisciplinary team (IDT) bedside discussions for high-risk patients, essentially replicating an Acute Care for Elderly (ACE) model. The typical ACE model of care emphasizes patient centered care, nurse driven prevention protocols, frequent interdisciplinary team rounds addressing common geriatric syndromes, and early discharge planning, and anticipation of care needs [8,9]. At the core of this model is an IDT, working together to improve care for older ED patients and impact care and outcome trajectories [10,11].

### 1.2. Securing Grant Support for Demonstration Project

Subsequent to the opening of the Geriatric ED, Mount Sinai received a Center for Medicare and Medicaid Innovation award as part of a clinical implementation demonstration program called GEDIWISE (Geriatric Emergency Department Innovations in Care through Workforce, Informatics, and Structural Enhancements) in collaboration with St Joseph’s Regional Medical Center in New Jersey and Northwestern Memorial Hospital in Illinois [12,13]. The three-year GEDI WISE program focused on implementing and demonstrating a new model of geriatric emergency care by providing a broader and more comprehensive range of services, assessments, and care coordination to address the complex needs of older adults in the ED. The goal of the GEDI WISE program was to provide a patient centered comprehensive approach to care, reduce avoidable inpatient hospital admissions and return visits to the ED. The program was a paradigm shift in the treatment and approach for older adults in EDs who are at risk for admission to the hospital [12]. 

**Figure 1:**
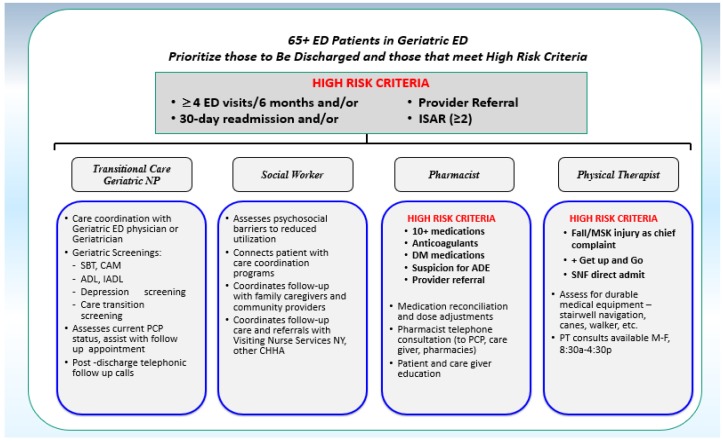
GEDI WISE IDT Algorithm: GEDI WISE: Geriatric Emergency Department Innovations in Care through Workforce, Informatics, and Structural Enhancements; ED: emergency department; ISAR: Identification of Seniors at Risk; NP: nurse practitioner; CAM: Confusion Assessment Method; ADE: adverse drug event; SNF: Skilled Nursing Facilities; PT: physical therapist. SBT: Short Blessed Test; ADL: Activities of Daily Living; IADL: Instrumental Activities of Daily Living; PCP: Primary Care Provider; CHHA: Certified Home Health Agency.

The GEDI WISE program provided additional resources specific to unique needs of older adults, improved staffing, and geriatric training for staff dedicated to enhancing geriatric care in the ED. The program supported and expanded the workforce to provide comprehensive evaluations to older patients in the ED facilitating critical decisions regarding hospital admission or ED discharge for complex cases. The geriatric IDT, including a transitional care nurse, social workers, pharmacists, and physical therapists, as shown in Figure 1, utilized algorithms of patient care and clinical care protocols tailored to treating older patients in the ED. An intervention design was created that could be used to explore ‘proof of concept’ of this model.

In this paper we will describe the creation of an ACE model in a Geriatric Emergency Department (including expanded IDT roles, focused geriatric assessments, clinical work flow) and how this model was implemented as part of the GEDI WISE intervention at The Mount Sinai Hospital. Preliminary program outcomes will be described. Previous patient outcome data has been published on the transitional care nursing role and social work intervention. 

## 2. Methods

### 2.1. Setting

Mount Sinai Hospital is a 1171 bed tertiary- and quaternary-care facility in New York City. The Mount Sinai Hospital Department of Emergency Medicine has an annual visit volume of 100,000 patients a year; those 65 years or older account for more than 15% of ED visits. Mount Sinai Hospital is also home to the Brookdale Department of Geriatrics and Palliative Medicine, one of the first freestanding academic geriatric departments in the country. 

### 2.2. Study Population

To assess impact of this Geriatric ED IDT intervention on programmatic as well as patient care outcomes, we conducted a retrospective chart review of patients 65 years and older who presented to the Mount Sinai Hospital Emergency Department (Mount Sinai ED) in New York City, New York between 1 January 2013 and 31 December 2015. During the study period there were 48,268 patient encounters (23,381 unique patients, 65 years and older) in the Mount Sinai ED. Of this cohort, 22,315 (12,328 unique patients, 65 years and older) met high-risk criteria as defined by Identification of Seniors at Risk (ISAR) ≥ 2. The study population consisted of the 6050 patients that received the intervention during the hours of the IDT staffing in the Mount Sinai ED. As seen in Table 1, 64.74% were female; 64.41% were 75 years of age or older; 91.04% had an Emergency Severity Index (ESI) score of 2 or 3 and 50.31% had an ISAR score ≥ 2. 

### 2.3. Program Description 

Mount Sinai’s GEDI WISE program essentially replicated a Geriatric ED ACE model with the goal of reframing the way care is provided for elders in the ED. GEDI WISE expanded an integrated and collaborative IDT workforce, and provided enhanced informatics to support focused geriatric assessments that improved clinical decision-making. There are four basic components introduced in this ACE model for the ED:

### 2.4. Dedicated Geriatric IDT Members and Roles

The Geriatric ED was staffed with an inter-professional team which included an emergency physician, emergency medicine residents, physician assistants, ED nurses, a transitional care geriatric nurse (TCN) and/or geriatric nurse practitioner (NP), social worker (SW), pharmacist (RX), physical therapist (PT), a consulting geriatrician and ED palliative care consultant, as shown in Table 2 and Table 3. The full staffing model and full time equivalent (FTE) support essentially included reframing and expanding the roles of the existing IDT members to optimize the care of older adults in the ED and the addition of a new clinical role for a transitional care nurse. All providers received additional training by the geriatrician in core geriatric education principles to meet the needs of this population. The role of the TCN/NP was to provide more focused geriatric assessments in the ED and facilitate the coordination required for complex transitional care planning of older adults being discharged from the ED. The role of PT was new to the ED, but therapists were able to incorporate standard assessments used in the inpatient hospital setting for evaluations in the ED. 

### 2.5. Geriatric Triage Assessments

Optimal geriatric care frequently requires a Comprehensive Geriatric Assessment (CGA) to address the medical, psychosocial, and functional complexities of this vulnerable patient population [6]. While CGAs can be time consuming, they are a critically important aspect of a coordinated care plan that aims to maximize overall health and improve patient outcomes. Assessments of older adults for geriatric specific syndromes were integrated into the clinical work flow of ED nurses at the point of triage and also during the primary nursing assessment. Enhanced electronic health record (EHR) support with discipline specific documentation templates for TCN, SW, RX, and PT helped capture these comprehensive assessments related to geriatric syndromes, cognitive and functional assessments, psycho social needs, and medication management. Patients over the age of 65 were screened with the Identification for Seniors at Risk (ISAR) score [15,16], Timed Get up and Go Fall risk assessment [17], and the brief Confusion Assessment Method (bCAM) for delirium [18]. Patients with ISAR score >2, fall risk, multiple prior ED visits, hospital admissions were considered to have met high-risk criteria.

### 2.6. Discipline Specific Geriatric Assessments for the ED

Patients who met high-risk criteria would subsequently receive IDT consultation through a collaborative identification process involving various disciplines (SW, PT, pharmacy, and nursing) and Geriatric Emergency Medicine providers. Additional criteria guided the specific IDT consults to assure focus on the highest risk patients, and to maximize impact and improve patient outcomes, as shown in Figure 1 and Table 3. Geriatric TCN/NP and social work assessments, for example, were frequently performed on high-risk patients prior to either hospital admission or discharge designation, and integrated recommendations from the ED provider’s clinical workup into the admission or discharge plans. The transitional care nurses (TCN) perform focused assessments including cognitive and functional screens to assist in the evaluation of the complex, older adults presenting with change in mental status, falls, functional decline, or delirium in the setting of the acute illness. They are equipped to make recommendations to focus further workup in ED, transitions of care, and evaluations during hospitalization if admitted. For patients who were discharged, the TCN helps to improve communication and facilitate care coordination across practice sites, ensure appropriate outpatient services to reduce unnecessary utilization, and assist with safe discharge and transition planning.

Similarly, the role of the SW in the ED setting is versatile and encompasses a multitude of complex psychosocial needs of this population. The social worker can help elderly individuals with psychosocial, medical, and/or financial challenges they may experience. As part of the care team, they are available to assist with assessments, offer counseling services which often deal with end-of-life issues, bereavement, elder abuse, and other concerns common in older adults. They are helpful identifying community resources for meals, transportation, and applying for medical benefits and other resources available to elders.

Pharmacy (RX) and physical therapy (PT) consults, on the other hand, had narrower criteria that focused their assessment primarily on high-risk patients who were to be discharged to home. For example, a pharmacy consult was triggered in patients taking 10 or more medications, or if their clinical presentation was associated with an adverse drug event (ADE). However, a pharmacy consult could also be triggered to guide real time treatment decisions after discussion during IDT rounds.

PT consults were triggered by a new fall, or musculoskeletal injury due to a fall, or to assess for a transition to a sub-acute rehabilitation facility. However, PT were also available to assess patients at the request of the ED team to ensure an evaluation for a safe discharge to home if needed. Geriatrician and palliative care providers were also consulted by the IDT clinical team for highest risk and complex cases when necessary. While the interdisciplinary team members created established criteria to guide assessments, consults could also be triggered simply by a recognized need that the patient could benefit from a more comprehensive evaluation. It was rare for assessments to be completed on patients who did not meet the need for an evaluation.

### 2.7. Incorporating the ACE Model into a Geriatric ED Clinical Work Flow

Clinical work flow is a crucial aspect of ED care. In an ED environment where efficiency and expedited workup is imperative, the IDT worked in parallel with the ED treatment team to identify complex clinical and psychosocial needs while assisting in the coordination of clinical evaluations and safe transitions for patients upon discharge from the ED. Patients received individual IDT consultations throughout the workday (8:00–20:00 Monday–Friday), and results were discussed with the entire team during designated IDT midday discussions. These IDT rounds were led by the emergency physician or geriatrician with the opportunity for all IDT members to present updates on cases and identify patients with specific issues requiring targeted consultations by the team. This dedicated time for daily rounds allowed the team members to collaborate and discuss high-risk cases, troubleshoot complex clinical and psychosocial issues, make use of inter-professional team members’ unique skill sets, develop comprehensive disposition plans with the goal of improving health care outcomes, intervene with community resources, and prevent avoidable admission. These discussions also provided a forum for inter-professional education, information exchange, and guidance on how to address common issues for future patients. The geriatric emergency provider was the point person for clinical issues or updates that came up after the IDT daily rounds and throughout the shift. Patients identified after the IDT rounds were discussed with individual consultants on an as-needed basis. Staff were encouraged to work collaboratively even after IDT rounds. All disciplines were available for consultations throughout the day.

### 2.8. Data Sources

Data on all ED visits at Mount Sinai Hospital between 1 January 2013 through 31 December 2015 were abstracted from the EPIC Electronic Medical Record and Mount Sinai Data Warehouse and included administrative and clinical data for all inpatient and ED visit encounters for patients 65 years and older.

### 2.9. IDT Consultation

Each encounter level consultation note entered by an interdisciplinary team member (i.e., TCN, SW, PT, pharmacist) was considered an indication of an assessment/intervention by the IDT during the ED visit encounter.

## 3. Results

There were 48,268 ED visit encounters during the study period made by 23,381 unique patients 65 years and older. Of the 22,315 who met high-risk criteria as defined by ISAR ≥ 2, 3321 (14.8%) of these received a GEDI WISE IDT consultation, representing 2606 unique patients. Of these, 1360 (40.90%) involved a geriatric RN/NP consultation, 2389 (71.9%) involved a social work consultation, 826 (24.8%) involved a pharmacy consultation, 616 (18.5%) involved a physical therapy consultation, and 82 (2.46%) involved consultations from all four disciplines (geriatric RN/NP, social work, pharmacy, and physical therapy), as shown in Table 4. Official consultations from Geriatrics and Palliative Care were requested as needed.

### 3.1. Assessments by Transitional Care RN/NP

Previous studies have demonstrated that individual geriatric assessments completed by the transitional care NP were associated with lower risk of admission, but greater risk of a 72 h ED revisit. Risk of any admission within 30 days of the index ED visit also remained lower for TCN patients thus indicating these patients were safely discharged during the index ED visit [19]. For patients being discharged from the ED, the transitional care RN or NP completed post-discharge phone calls to ensure proper follow up with outpatient providers, consultants, and ensure seamless care coordination after an ED visit.

### 3.2. Assessments by Social Work

The most commonly requested assessment for older adults in the ED was an evaluation by the social worker. Seventy percent of the study population had contact with a SW, however the interventions varied in intensity. The majority of interventions facilitated safe transitions to home for patients who were unlikely to be admitted (i.e., home safety assessment, transportation assistance, or access to visiting nurses and community resources) [20]. The SW assessment was also crucial in exploring the patient’s psychosocial needs and connecting patients with the proper community resources so patients could safely return home and avoid unnecessary admissions.

### 3.3. Assessments by Pharmacy

Older adults with multiple co-morbidities, are more likely to be prescribed a greater number of medications and hence at greater risk for adverse drug interactions. Pharmacy consults in the study were initiated in two ways: (1) to help facilitate a safe discharge especially in patients with polypharmacy or taking a high-risk medication; or (2) pharmacists also assessed admitted patients who were boarding in the ED. As a result, there were a disproportionate number of consults by pharmacy associated with admissions, as they frequently completed medication reconciliation for the admitted patients.

### 3.4. Assessments by Physical Therapy

Patients who met criteria for PT evaluation were screened for home safety, the need for assistive devices, or assistance in safe transition planning. In addition, PT evaluation facilitated transitions to Skilled Nursing Facilities (SNF) or sub-acute rehabilitation (SAR) from the ED for eligible patients. Evaluations by PT helped ensure safe disposition plans for patients with functional limitations. Despite PT evaluation in the ED, however, many SNF or SAR eligible patients were admitted if they required a 72 h hospitalization to satisfy the Centers for Medicare and Medicaid Services (CMS) requirements.

### 3.5. Assessments by More Than One Discipline

**Figure 2:**
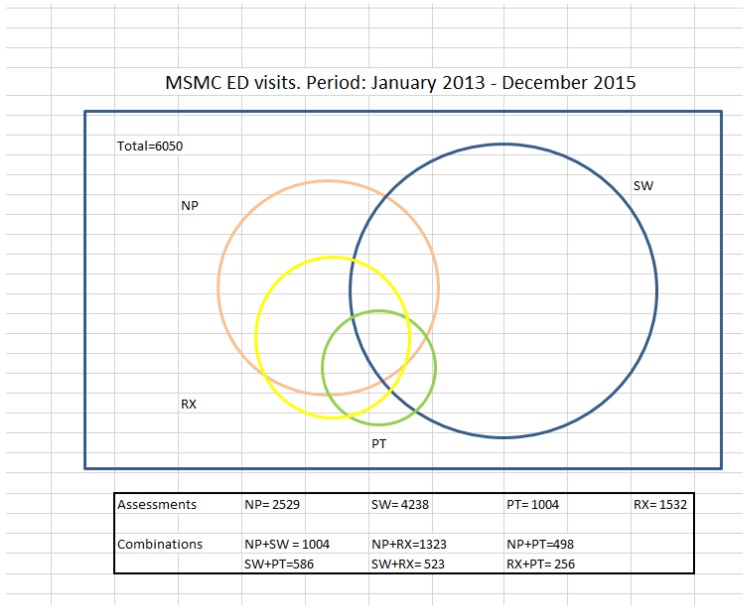
Mount Sinai Medical Center ED visits. Period: January 2013–December 2015. Blue = SW; Orange = NP; Yellow = Rx; Green = PT.

Many individuals had multiple consultations during the same ED visit. The most common combinations of shared assessment included NP and pharmacist or NP and SW, as shown in Figure 2. Team members could decide to see patients together, or individually, and then make final recommendations once information was gathered by the various disciplines. Furthermore, clinical decision-making and care planning were enhanced by the multiple sources of evaluation. Figure 2 highlights the overlap in collaborative assessments per discipline.

### 3.6. Additional Criteria Guided the IDT Consults

In order to focus interventions on the highest risk patients to maximize impact and improve patient outcomes, specific criteria were created to trigger consults. As described above, geriatric RN/NP and social work assessments were broader in focus and performed primarily on high-risk patients prior to admission or discharge designation. Pharmacy and physical therapy consults, on the other hand, had a narrower and specific focus, assessing only high-risk patients that were confirmed for discharge to home, as shown in Figure 1, Table 2 and Table 3. While established criteria were created to guide the assessments by the interdisciplinary team members, consultations could be requested by a clinical provider simply by a recognized need that the patient could benefit from a more comprehensive evaluation. As seen in Table 4, approximately 6–30% of IDT assessments were completed on patients who did not necessarily meet sole criteria of ISAR ≥ 2, but met criteria for a discipline-specific intervention.

### 3.7. Variation in Staffing and Community Resources

Staffing resources varied throughout the study period. The full staffing model could not be fully matched to patient arrivals at the Mount Sinai ED which receives patients 24 h a day, 7 days a week, 365 days per year. The IDT staffing hours were designed to match ED arrival patterns of patients >65 years old as well as the availability of community resources (i.e., Skilled Nursing Facilities, visiting nurse, and other community resources) for coordination and admission, typically Monday through Friday 9:00–16:00. The staffing and hours in this model included: geriatric RN/NP 8:00–20:00, SW 8:00–20:00 onsite full coverage, 20:00–8:00 offsite SW limited coverage for transportation and elder abuse cases. Physical therapy provided 8:00–16:00 coverage and a pharmacist available for 8:00–20:00 coverage. Geriatric medicine and palliative consults were available throughout the day as needed, for patients who were not in ED during the time of daily IDT rounds.

## 4. Discussion

For older adults, a visit to the ED and a subsequent hospitalization is considered a sentinel event, often associated with a decline in the patient’s health status and quality of life. An admission to the hospital should therefore be prevented whenever possible for this population to avoid the greater risk for adverse outcomes, such as delirium, iatrogenic infections, medication errors, adverse drug events, and functional decline, which in turn lead to more complex transitions to post-acute care, rehabilitation, or nursing home placement [6,21].

Older adults who present to an emergency department (ED) generally have more complex medical conditions with complicated care needs and are at high risk for preventable adverse outcomes during their ED visit. The ED is uniquely positioned to alter care trajectories and prevent complications that significantly affect the patients’ outcomes. The addition of an interdisciplinary team and enhanced geriatric assessments in the ED can guide clinical decisions and prevent avoidable hospital admissions and impact care outcomes for this vulnerable population.

The ACE model that we describe here addresses a growing need for older adults in the ED. Our project demonstrated the feasibility of implementing principles of an ACE practice in an emergency department setting. Integrating geriatric assessments into the ED clinical work flow to identify specific geriatric syndromes, such as dementia, delirium, functional status, frailty, or polypharmacy, allows all providers to address significant patient care needs and make better clinical decisions. Incorporating an interdisciplinary team model into the Geriatric Emergency Department also allows providers to better address the potential gaps in the care of older patients, specifically in cognitive assessment, medication management, and safe transitional care planning [22].

In this care model, the focus is on identifying patients who have the highest needs, reducing avoidable admissions, and decreasing ED revisits by developing safe, realistic, and seamlessly coordinated discharge plans. In order to implement this model in a busy ED setting, IDT assessments and consultations were prioritized for patients who met specific criteria and could potentially be discharged from the ED. Social work evaluations were the most commonly requested interventions, followed by NP, pharmacist, and then PT. This was most likely due to the fact the SW and NP assessments covered a broader scope of issues, compared to the more limited criteria for pharmacy and PT consultation evaluations.

The key aspect of programmatic implementation included securing support from hospital leadership. Additional staffing underwritten by a unique grant funding opportunity was critically important and allowed the ED to leverage and enhance existing resources with augmentation in staffing, building of care protocols, assessment templates, and criteria to identify at risk patients, and geriatric-focused training and education. This model illustrates how the realignment and addition of dedicated resources with an interdisciplinary team can enhance the care for older adults in the ED.

The collaboration by IDT disciplines allows for more comprehensive evaluation to guide informed clinical decision-making and better align with quality of care, patient safety, and health system priorities. By focusing on the patients who have the highest need these programs are able to develop safer, feasible, and seamlessly coordinated care plans for patients that could be discharged and reduce their risk for hospital admission.

## 5. Conclusions

This innovative model of geriatric emergency care we describe reframes the traditional model of ED care to address these unique care needs of older adults in the ED. In this model we were able to able to create clinical protocols to identify high risk older adults, incorporate IDT assessments to better guide clinical decision making, and facilitate transitional care after an ED visit.

Further publications from the GEDI-WISE team will review the impact of all aspects of this model on patient outcomes such as discharge dispositions, readmission rates, hospital LOS for those admitted, final post-acute care destination and cost savings. Analysis of these outcomes will identify which combinations of IDT interventions are most effective in improving emergency care for older adults.

The importance of this new approach to geriatric emergency care is already widely recognized. In 2014, the American College of Emergency Physicians, American Geriatrics Society, Emergency Nurses Association and the Society for Academic Emergency Medicine created the Geriatric Emergency Department Guidelines with descriptions of requirements for structure, screening protocols, staff education and community resources to meet the criteria to qualify as a Geriatric Emergency Department [22]. Subsequently, in January 2018, the American College of Emergency Physicians, with support from the Gary and Mary West Health Institute and the John A. Hartford Foundation, launched an accreditation program for geriatric emergency departments that have met certain quality standards [23,24,25,26]. In May 2018, The Mount Sinai Hospital received ACEP Geriatric Emergency Department accreditation, one of the first Level 1 GEDs in the United States.

## Figures and Tables

**Table 1:** Acute Care for Elderly (ACE) Model in ED Demographics.

N = 6050	Frequency	Percentage (%)
**Gender**		
Female	3917	64.74
Age		
65–74	2153	35.59
75–84	2096	34.64
>85	1801	29.77
Emergency Severity Index (ESI) *		
1	23	0.38
2	1274	21.06
3	4234	69.98
4	487	8.05
5	18	0.30
Not Documented	14	0.23
Identification of Seniors at Risk (ISAR) ** (Score ≥ 2 Is Considered High Risk)		
0	316	5.22
1	1531	25.31
2	1139	18.83
3	947	15.65
4	816	13.49
5	305	5.04
6	114	1.88
Not Documented	882	14.58
**Race/Ethnicity**		
Native American	7	0.11
Asian	90	1.48
Pacific Islander	1	0.001
African American	1847	30.5
White	1532	25.3
Hispanic	2080	34.3
Unknown	493	8.14

* The Emergency Severity Index (ESI) is a five-level emergency department (ED) triage algorithm that provides clinically relevant stratification of patients into five groups from 1 (most urgent) to 5 (least urgent) on the basis of acuity and resource needs. The Agency for Healthcare Research and Quality (AHRQ) funded initial work on the ESI [14]. ** ISAR: Identification of Seniors at Risk Score. The ISAR is a six-item risk-screening tool for elderly patients seen in the ED. The ISAR is a self-report screening tool composed of six simple “yes/no” items, related to functional dependence, recent hospitalization, impaired memory and vision, polypharmacy. The total scale range is from 0 to 6, as each item is scored 1 if the patient reports having the problem and 0 if not. The ISAR was developed and validated in EDs in Canada in 1999, to identify elderly patients at risk of adverse outcomes [15,16].

**Table 2:** GEDI WISE Interdisciplinary team (IDT) members and roles. FTE: full time equivalent.

IDT Members	Clinical Roles
Geriatric ED Physician (1 FTE) Geriatric ED Resident/Physician Assistant (2 FTE)	Acute medical management
ED Nurse (2 FTE)	Performed universal triage screening for functional decline, delirium, fall assessment.
Transitional Care Nurse or Nurse Practitioner (TCN/NP) (2 FTE)	Comprehensive Geriatric Assessments: cognitive, functional, behavioral, nutritional, incontinence, medication management, pain management, vision and hearing, healthcare access, discharge planning, advanced care planning, social support to identify high-risk patients and provide support for social and functional needs. Care Transition Discharge follow up phone call: reviewed clinical status and discharge instructions, medications, knowledge of red flags, coordinated follow up appointments to ensure safe care transitions.
Geriatric ED Social Worker (1–2 FTE)	Psychosocial Assessment: family/social situation, behavioral, cognitive, functional, home safety, elder abuse, financial, medications, durable medical equipment, health literacy, community resource referrals, caregiver strain, advance care planning
Geriatric ED Pharmacist (1 FTE)	Geriatric Pharmacy Assessment: Polypharmacy, medication management, medication education, identification of inappropriate medications
Geriatric ED Physical Therapist (0.25 FTE)	Fall assessment, durable medical equipment (DME) recommendations; rehab referrals
Geriatrician (0.25)	Consultant, geriatric-focused education
Geriatric ED Palliative Care Consultant (0.5 FTE)	Consultant, symptom management, Goals Of Care, transition planning

**Table 3:** GEDI WISE IDT Assessments.

IDT Members	Clinical Assessments
Transitional Care Nurse /(TCN/NP)	Functional/cognitive assessments, referral to PCP, focused assessments (SBT, CAM, Katz Functional assessment, depression screen, caregiver burden, nutrition)
Social Worker (SW)	Transportation, home care, Visiting Nurse referrals, PT referral, hospice, SNF placement, Elder abuse screen, Meals on Wheels, community resources
Geriatric Pharmacist (RX)	Medication reconciliation; medication changes, high risk medication intervention, (Beers tool, adverse drug events, triggered for high risk medications, i.e., anticoagulants)
Physical Therapy (PT)	Functional assessments, durable medical equipment requirements and supplies, referral to skilled nursing facilities, sub-acute rehabilitation and home rehabilitation

**Table 4:** GEDI WISE: an inter-professional Acute Care for the Elderly (ACE) model for older adults in the ED.

**IDT Interventions**	**Study Population Details**	
	Cohort Time Period: 1 January 2013 to 31 December 2015	
	44,268 patients over the age of 65 in ED	
	22,315 patients over age of 65 considered High-risk (ISAR ≥ 2)	
Total Number of IDT Interventions	6050	
Total Number of IDT Interventions on high-risk patients: (ISAR ≥ 2)	3321	
**GEDI WISE IDT Consultation Intervention**	**Total N (Percent)**	**Total (N) for High-Risk (Percent)**
Transitional Care Nurse Intervention (TCN/NP)	2529 (41.85)	1360 (40.9)
Social Worker (SW)	4238 (70.08)	2389 (71.9)
Pharmacist (RX)	1532 (25.3)	826 (24.8)
Physical Therapy (PT)	1004 (18.5)	616 (16.5)

N—IDT assessments.
